# The factors present in regenerating muscles impact bone marrow-derived mesenchymal stromal/stem cell fusion with myoblasts

**DOI:** 10.1186/s13287-019-1444-1

**Published:** 2019-11-21

**Authors:** Paulina Kasprzycka, Karolina Archacka, Kamil Kowalski, Bartosz Mierzejewski, Małgorzata Zimowska, Iwona Grabowska, Mariusz Piotrowski, Milena Rafałko, Agata Ryżko, Aliksandra Irhashava, Kamil Senderowski, Magdalena Gołąbek, Władysława Stremińska, Katarzyna Jańczyk-Ilach, Marta Koblowska, Roksana Iwanicka-Nowicka, Anna Fogtman, Mirosław Janowski, Piotr Walczak, Maria A. Ciemerych, Edyta Brzoska

**Affiliations:** 1 Department of Cytology, Faculty of Biology, University of Warsaw, Miecznikowa 1 St, 02-096 Warsaw, Poland; 2 Laboratory of Systems Biology, Faculty of Biology, University of Warsaw, Pawinskiego 5a St, 02-106 Warsaw, Poland; 3 Laboratory of Microarray Analysis, Institute of Biochemistry and Biophysics, Polish Academy of Sciences, Pawinskiego 5a St, 02-106 Warsaw, Poland; 4 Russell H. Morgan Department of Radiology and Radiological Science, Division of MR Research, The Johns Hopkins University School of Medicine, Baltimore, MD 21205 USA; 5 Cellular Imaging Section and Vascular Biology Program, Institute for Cell Engineering, The Johns Hopkins University School of Medicine, Baltimore, MD 21205 USA; 6 NeuroRepair Department, Mossakowski Medical Research Centre, Polish Academy of Sciences, Pawinskiego 5 St, 02-106 Warsaw, Poland; 7 Department of Neurosurgery, School of Medicine, Collegium Medicum, University of Warmia and Mazury, 10-719 Olsztyn, Poland; 8 Institute for Cell Engineering, Cellular Imaging Section, The Johns Hopkins University School of Medicine, Baltimore, MD 21205 USA

**Keywords:** BM-MSC, Fusion, IGF-1, IL-4, IL-6, Myogenic differentiation, SDF-1

## Abstract

**Background:**

Satellite cells, a population of unipotent stem cells attached to muscle fibers, determine the excellent regenerative capability of injured skeletal muscles. Myogenic potential is also exhibited by other cell populations, which exist in the skeletal muscles or come from other niches. Mesenchymal stromal/stem cells inhabiting the bone marrow do not spontaneously differentiate into muscle cells, but there is some evidence that they are capable to follow the myogenic program and/or fuse with myoblasts.

**Methods:**

In the present study we analyzed whether IGF-1, IL-4, IL-6, and SDF-1 could impact human and porcine bone marrow-derived mesenchymal stromal/stem cells (hBM-MSCs and pBM-MSCs) and induce expression of myogenic regulatory factors, skeletal muscle-specific structural, and adhesion proteins. Moreover, we investigated whether these factors could induce both types of BM-MSCs to fuse with myoblasts. IGF-1, IL-4, IL-6, and SDF-1 were selected on the basis of their role in embryonic myogenesis as well as skeletal muscle regeneration.

**Results:**

We found that hBM-MSCs and pBM-MSCs cultured in vitro in the presence of IGF-1, IL-4, IL-6, or SDF-1 did not upregulate myogenic regulatory factors. Consequently, we confirmed the lack of their naïve myogenic potential. However, we noticed that IL-4 and IL-6 impacted proliferation and IL-4, IL-6, and SDF-1 improved migration of hBM-MSCs. IL-4 treatment resulted in the significant increase in the level of mRNA encoding CD9, NCAM, VCAM, and m-cadherin, i.e., proteins engaged in cell fusion during myotube formation. Additionally, the CD9 expression level was also driven by IGF-1 treatment. Furthermore, the pre-treatment of hBM-MSCs either with IGF-1, IL-4, or SDF-1 and treatment of pBM-MSCs either with IGF-1 or IL-4 increased the efficacy of hybrid myotube formation between these cells and C2C12 myoblasts.

**Conclusions:**

To conclude, our study revealed that treatment with IGF-1, IL-4, IL-6, or SDF-1 affects BM-MSC interaction with myoblasts; however, it does not directly promote myogenic differentiation of these cells.

## Background

Satellite cells are skeletal muscle-specific unipotent stem cells, retained in a quiescent state, and characterized by the expression of PAX7 transcription factor. Their role in skeletal muscle regeneration is well characterized [1–3]. As a result of muscle injury, the numerous cytokines and growth factors are released by damaged muscle fibers, as well as endothelial cells, fibroadipogenic progenitors (FAP), fibroblasts, and inflammatory cells [4–6]. Some of these factors such as hepatocyte growth factor (HGF), insulin-like growth factor-1 (IGF-1), fibroblast growth factor-2 (FGF-2), and tumor necrosis factor-α (TNF-α) activate signaling pathways controlling cell cycle re-entry of satellite cells and enable their activation [2, 7]. As a result, satellite cells start proliferation and either generate myogenic progenitors/myoblasts, which undergo further differentiation, or return to the quiescent state and renew the satellite cell pool. The proliferation and differentiation of satellite cells and myoblasts are regulated inter alia by myogenic regulatory factors (MRFs) such as MYOD1, MYF5, myogenin (MYOG), and MFR4 (also known as MYF6) [8]. The activated satellite cells express both *MYOD1*, which induces the expression of genes encoding factors regulating cell cycle and *MYOG*, which mediates cell cycle exit and expression of genes necessary for differentiation into post-mitotic myocytes expressing *MRF4*. Then, in regenerating muscle, the fusion of myocytes leads to myotube and myofiber formation. Many intrinsic and extrinsic factors regulate the differentiation of myoblasts participating in skeletal muscle regeneration. In the present study we focused on the impact of IGF-1, interleukin-4 (IL-4), interleukin-6 (IL-6), or stromal derived factor-1 (SDF-1) on human and porcine bone marrow-derived mesenchymal stromal/stem cell (hBM-MSCs and pBM-MSCs) differentiation and fusion with myoblasts. These factors were selected on the basis of their role in embryonic myogenesis and skeletal muscle regeneration.

IGF-1, by interacting with its receptor—IGF-1R, activates at least two pathways involved in determination of cell fate, i.e., ERK1/ERK2 MAPK (extracellular signal-regulated protein kinases 1/extracellular signal-regulated protein kinases 2 mitogen-activated protein kinase) pathway which promotes cell proliferation [9] and p38 MAPK pathway which stimulates satellite cell differentiation [10–12]. IGF-1 can also induce phosphoinositide-3-kinase (PI3K)-dependent pathways, like PI3K/AKT (protein kinase B), PI3K/AKT/mTOR (mammalian target of rapamycin kinase), or PI3K/AKT/GSK3 (glycogen synthase kinase 3). Among the effects of PI3K/AKT action is the induction of the expression of *MEF2* and MRFs, such as *MYOD1* and *MYOG* [13]. Next, it was shown that stimulation of PI3K/AKT/GSK3 and PI3K/AKT/mTOR pathways by IGF-1 induces myotube hypertrophy by phosphorylation of downstream targets, such as p70S6 kinase, 4E-BP1, or eIF2, which are directly involved in the regulation of translation [14, 15]. The effect of IGF-1 was also tested in *mdx* mice. IGF-1 overexpression in mice skeletal muscles resulted in the reduction of myofiber atrophy, necrosis, and fibrosis [16, 17]. IGF-1 not only impacts myogenesis per se but also enhances the recruitment of stem cells from the bone marrow to the sites of muscle injury [18].

The next factor selected by us, i.e., IL-4, is a pleiotropic cytokine first described as a B cell stimulatory factor [19]. It also modulates the activity of other cell types, i.e., T cells and mast cells [20, 21]. The action of IL-4 can be transduced by two types of receptors: type I consisting of the IL-4Rα and γC subunits—expressed by hematopoietic cells, and type II consisting of the IL-4Rα and IL-13Rα1 subunits—expressed by non-hematopoietic cells, including myogenic cells, i.e., myoblasts, both in mouse and human [22]. In 2003, IL-4 was described as the myogenesis regulator engaged in recruiting mononuclear myoblasts to the newly formed myotubes and enabling their growth. Mice lacking IL-4 or IL-4Rα were characterized by a decreased number of nuclei present in myofibers as well as an increased proportion of smaller myofibers and a decreased proportion of larger ones [23]. Next, IL-4 was shown to promote migration of myogenic cells both in vitro and in vivo, i.e., during muscle regeneration, by increasing *Itagb1* and *Itagb3* expression [24]. IL-4 was also shown to play an important role in muscle growth during postnatal development. Mice lacking serum response factor (SRF), a transcription factor regulating expression of different muscle-specific genes such as muscle creatinine kinase and dystrophin, were characterized by strong downregulation of *Il-4* expression and—as a consequence—impaired recruitment of myoblasts to myofibers, retarded postnatal muscle growth, and decreased muscle mass [25]. IL-4 possibly influences the expression of proteins localized on myogenic cell surface, similarly as it was described for smooth muscles [26], lymphocytes B [27], fibroblasts [28], and macrophages [29], but the precise mechanism of IL-4 action in myogenic cells is not known yet.

IL-6 is another pleiotropic cytokine classified both as pro- and anti-inflammatory protein. It is produced by many cell types, such as activated macrophages, vascular endothelial cells, and fibroblasts [30, 31] and locally in skeletal muscles where its level is related to the glycogen level, i.e., when the level of glycogen decreases the IL-6 production increases [32]. IL-6 is also secreted in vitro by human primary myoblasts and mouse C2C12 myoblasts [33, 34] and promotes their differentiation. Silencing of *Il-6* gene expression in myoblasts leads to the downregulation of muscle-specific genes—*α-actin* and *Myog* [35]. Next, mice lacking IL-6 were characterized by defective muscle growth resulting from impaired proliferation and migration of satellite cells which indicates the role of this cytokine in satellite cell-mediated hypertrophy [36]. Thus, IL-6 is a significant mediator of both cell proliferation and myogenic differentiation and through these effects plays an important role in skeletal muscle growth and regeneration.

The last factor selected by us—stromal derived factor-1 (SDF-1, also known as CXCL12)—is a CXC chemokine, which binds to CXCR4 or CXCR7 receptors or CXCR4/CXCR7 heterodimers [37–39]. SDF-1 binding to CXCR4 leads to a number of cellular events, such as inhibition of adenylate cyclase, PI3K, and Rho pathways as well as ERK1/ERK2 MAPK and p38 MAPK independently of G-proteins [40]. The SDF-1 expression increases in injured tissues as it was shown for skeletal muscles [41–43] where SDF-1 is mainly produced by damaged myofibers [43]. We previously observed that SDF-1 treated muscles regenerated more efficiently due to CXCR4^+^ and CD34^+^ cell mobilization [42, 44]. Unquestionably, SDF-1 promotes the migration of myoblasts [42, 45–48] by inducing changes in actin organization via activation of FAK (focal adhesion kinase), CDC42 (cell division control protein 42), and RAC-1 (Ras-related C3 botulinum toxin substrate 1) [47]. Importantly, SDF-1 treatment of myoblasts also increased the expression of the tetraspanin CD9 that plays a crucial role in cell migration and fusion [49] as well as led to MYHC accumulation and increased myotube formation [50]. Interestingly, the inhibition of *Myog* and *MYHC* expression was also reported for SDF-1-treated myoblasts [43], which stays in contrast to our results showing no significant impact of SDF-1 on neither myoblast proliferation nor MRF expression nor fusion [42].

On the basis of the abovementioned information, we hypothesized that factors involved in the regulation of myogenesis and skeletal muscle regeneration could impact the myogenic identity, migration, and fusion of human and porcine BM-MSCs with mouse myoblasts. It was shown previously that hBM-MSCs characterized by CD146 expression are not able to undergo spontaneous myogenic differentiation [51]; thus, these cells do not present naïve myogenic potential. However, it was also documented that in the presence of exogenous myoblasts, BM-MSCs can fuse with them, although with low efficiency [52]. Moreover, modification of BM-MSCs by *Pax3*, *β-catenin*, or *NICD* (Notch intracellular domain) overexpression can reprogram them to start myogenic differentiation and fuse to form myotubes [53–56]. In our previous study, we showed that interactions of bone marrow isolated stem cells with satellite cell niche can enhance their myogenic identity [57]. Thus, in the current study, we analyzed the influence of IGF-1, IL-4, IL-6, or SDF-1 on the BM-MSC proliferation, migration, myogenic potential, and fusion in vitro.

## Methods

### BM-MSC culture

Human BM-MSC commercial cell line was obtained from Lonza (Lonza, PT-2501). Porcine BM-MSCs were isolated from transgenic porcine embryos which constitutively expressed green fluorescent protein (GFP). Both human and porcine BM-MSCs were cultured in high-glucose DMEM (Dulbecco’s modified Eagle’s medium, Invitrogen) supplemented with 15% FBS (fetal bovine serum, Invitrogen) and 10 μg/ml gentamycin (Sigma Aldrich) in 5% CO_2_ at 37 °C. Culture medium was replaced every 2 days, and cells were passaged after reaching confluency. For further analysis, cells from passages 4–9 were used. Depending on the type of analysis, cells were collected and frozen (for further RNA isolation) or cultured on cover slides covered with a 1% gelatin solution and fixed with a 3% PFA (for protein immunolocalization).

### BM-MSC treatment or pre-treatment with IL-4, IL-6, IGF-1, or SDF-1

Human and porcine BM-MSCs were plated on 60-mm wells at a density of 3 × 10^3^ cells per 1 cm^2^ and cultured in high-glucose DMEM supplemented with 15% FBS and 10 μg/ml gentamycin in 5% CO_2_ at 37 °C for 3 days. At the fourth day of culture, the so-called proliferating medium, i.e., high-glucose DMEM containing 15% FBS and 10 μg/ml gentamycin (PM), or differentiating medium, i.e., high-glucose DMEM containing 10% FBS, 10% horse serum, and 10 μg/ml gentamycin (DM) supplemented with 50 ng/ml IGF-1 or IL-4 or IL-6 or SDF-1 [recombinant human IGF-1 protein ab155614 (Abcam); recombinant human IL-4 protein ab83686 (Abcam); recombinant human IL-6 protein ab119444 (Abcam); recombinant human SDF-1 alpha protein ab73461 (Abcam)], was added to cells. From that moment, PM or DM containing selected factors was replaced every day, for the next 7 days. Such protocol allowed to keep the constant concentration of analyzed factors. To follow the number of cells in culture, they were counted every day from days 1 to 7. The cells were washed with PBS, trypsinized, suspended in a culture medium, and counted in a hemocytometer. Cells treated for 7 days were also collected and frozen in − 80 °C or fixed with 3% PFA for further analyses. In another set of experiments, BM-MSCs were transiently treated with selected factors. Thus, BM-MSCs were cultured for 3 days in control PM and then the medium was changed to PM supplemented with 50 ng/ml of either IGF-1 or IL-4 or IL-6 or SDF-1 for 3 days. Next, the medium was changed to control PM and pre-treated hBM-MSCs were analyzed (including cell counting) after 1, 3, and 7 days of culture.

### Co-culture of human and porcine BM-MSCs and C2C12 myoblasts

C2C12 myoblasts, human BM-MSCs, and porcine BM-MSCs were separately cultured in high-glucose DMEM supplemented with 15% FBS and 10 μg/ml gentamycin in 5% CO_2_ at 37 °C. Then, each type of cells was trypsinized, washed, and suspended in the medium. Next, 25 × 10^3^ of BM-MSCs and 25 × 10^3^ of C2C12 myoblasts were plated together and co-cultured in 2 ml of high-glucose DMEM supplemented with 10 μg/ml gentamycin and 15% FBS (PM) or 10% FBS and 10% HS (DM). For the cell treatments, PM and DM were supplemented with 50 ng/ml IGF-1, IL-4, IL-6, or SDF-1. From that moment, culture medium (PM or DM) containing one of the factors was replaced every day for the next 7 days which allowed to keep the required concentration of analyzed factors. After 7 days of treatment, cells were collected and frozen or fixed with 3% PFA for further RNA analyses or immunostaining. In pre-treated co-cultures, the BM-MSCs were cultured in the presence of 50 ng/ml IGF-1, IL-4, IL-6, or SDF-1 in PM for 3 days. Then, the 25 × 10^3^ of pre-treated BM-MSCs and 25 × 10^3^ of C2C12 myoblasts were plated together and co-cultured in 2 ml of high-glucose DMEM supplemented with 15% FBS and 10 μg/ml gentamycin (PM) for 1, 3, and 7 days when they were collected and frozen or fixed with 3% PFA for further RNA analyses or immunostaining.

### The cell migration assay

The 1 × 10^4^ hBM-MSCs were plated in the culture dish (35 mm) and cultured until they reached 90% of confluency. Linear scratches were made in cell sheets using a 200μl pipette tip. The culture medium was replaced with a fresh control culture medium or the one containing either SDF-1, IGF-1, IL-4, or IL-6 in concentrations of 10, 50, and 100 ng/ml. The scratch area was photographed directly after the scratch and after 6 h and 12 h. Next, the area of the scratch was measured for control and each experimental group. Results were presented as relative mobility of treated cells compared to control, i.e., untreated cells and represent four independent experiments with three technical repeats each.

### Quantitative reverse transcriptase real-time PCR

RNA was isolated from human and porcine BM-MSCs, mouse C2C12 myoblasts, and co-cultures of C2C12 myoblasts with either hBM-MSCs or pBM-MSCs using High Pure Isolation Kit (Roche Applied Science). Three independent cell cultures were used per each experiment). The cDNA was obtained using RevertAid First Strand cDNA Synthesis Kit (Thermo Fisher Scientific) in accordance with the manufacturers’ protocols. Then, using TaqMan® Gene Expression Master Mix and TaqMan assays (Thermo Fisher Scientific), the quantitative real-time PCR was performed. Hypoxanthine phosphoribosyltransferase 1 (*HPRT1*) was used as the reference gene. All reactions were performed in technical duplicates. The conditions of RT-qPCR were as follows: reverse transcription: 25 °C for 10 min, 42 °C for 60 min, 85 °C for 5 min; qPCR: 50 °C for 2 min, template denaturation 95 °C for 10 min, 45 cycles of 95 °C for 15 s and 60 °C for 60 s. Threshold-cycle (Ct) values of the analyzed amplicons were determined with LightCycler®480 Software (Roche Applied Science). Expression levels were calculated with the 2^-(ΔΔCt)^ formula using a relative quantification tool in LightCycler® 480 Software [58]. The samples were compared to non-treated cells (NT). The Ct over 32 cycles were not analyzed. TaqMan assays: hADAM9 (Hs00177638_m1); mADAM9 (Mm00475770_m1), hCD9 (Hs00233521_m1); hCDH15 (m-cadherin) (Hs00170504_m1); hCXCR4 (Hs00607978_s1), hCXCR7 (Hs00664172_s1), hDES (desmin) (Hs00157258_m1); hHPRT1 (Hs02800695_m1); mHPRT1 (Mm03024075_m1); hIGF-1 (Hs01651089_g1); hIGFR (Hs00609566_m1); hIL-4 (Hs00598625_m1); hIL-4R (Hs00965056_m1); hIL-6 (Hs00174131_m1); hIL-6R (Hs01075664_m1); hMYF5 (Hs00929416_g1); hMYOD1 (Hs00159528_m1); hMYOG (myogenin) (Hs01072232_m1); mMYOG (myogenin) (Mm00446194_m1); hMYH3 (MyHC3) (Hs01074230_m1); mMYH3 (MyHC3) (Mm01332463_m1); hNCAM1 (Hs00941830_m1); hSDF-1 (Hs00664172_s1); hVCAM (Hs01003372_m1).

### Microarray analysis

Human BM-MSCs were cultured in high-glucose DMEM supplemented with 15% FBS and 10 μg/ml gentamycin and non-treated or treated with IL-4 (50 ng/ml) for 7 days. Total RNA was isolated using the mirVana Isolation Kit (Thermo Fisher Scientific). Next, its integrity was checked with 2100 Bioanalyzer (Agilent Technologies) using RNA 6000 NAno LAb Chip kit (Agilent Technologies). All RNA samples had integrity number above 8.5. One hundred nanograms of total RNA for each sample was biotin labeled with the TargetAmpTM-Nano Labeling Kit for Illumina Expression BeadChip (Epicentre Biotechnologies). Labeled RNA was purified with RNeasy MinElute Cleanup Kit (Qiagen) and hybridized onto MouseRef-8 v2.0 Expression BeadChip (illumina) according to the manufacturer’s instructions. Arrays were scanned with a HiScanSQ System (illumina). Raw data were imported to GenomeStudio (illumina), and the average signal intensities were analyzed in Partek Genomic Suite (Partek, Inc.) v. 6.6 after quantile normalization and Log2 transformation. Qualitative analysis was performed, e.g., principal component analysis, in order to identify outliers and artifacts on the microarray. After the quality check, the two-way ANOVA (analysis of variance) model by using Method of Moments was performed on the data and lists of significantly and differentially expressed genes between biological variants [with the cutoff values: *p* value < 0.05, (1.3 ≥ fold change ≥ 1.33)] were created. Fisher’s least significant difference (LSD) was used as the contrast method to compare non-treated hBM-MSCs vs treated with IL-4 hBM-MSCs. Unsupervised hierarchical clustering was performed on the selected lists in order to find genes and samples with similar profiles. Gene networks were created by interposing the results onto the database of Ingenuity containing information about gene functions with the use of Ingenuity Pathway Analysis tool.

### Protein immunolocalization

Human and porcine BM-MSCs, mouse C2C12 myoblasts, and co-cultures of BM-MSCs and C2C12 myoblasts were fixed with 3% PFA in PBS, washed in PBS, permeabilized by incubation in 0.05% Triton X100 in PBS, washed in PBS, then incubated in 0.15% glycine in PBS, and blocked in 3% BSA in PBS. Then, cells were incubated overnight with primary antibodies diluted 1:100 in 3% BSA at 4 °C. Next, cells were washed with PBS and incubated with fluorochrome-conjugated secondary antibodies diluted 1:200 in 3% BSA in PBS for 2 h at room temperature. After washing in PBS, cells were incubated with DRAQ5 (Biostatus) diluted 1:1000 in PBS for 5 min, washed in PBS, and mounted with Dako Cytomation Fluorescent Mounting Medium. Fluorescence was analyzed using confocal microscopy (LSM 500, Zeiss) and ZEN application (Zeiss). Antibodies used were: anti-human nuclear antigen antibody [235-1] (Abcam); anti-myosin (Skeletal), antibody produced in rabbit (Sigma-Aldrich); donkey anti-mouse IgG (H+L) highly cross-adsorbed secondary antibody, Alexa Fluor 488 (Thermo Fisher Scientific Scientific); and donkey anti-rabbit IgG (H+L) highly cross-adsorbed secondary antibody, Alexa Fluor 594 (Thermo Fisher Scientific Scientific).

### Giemsa–May–Grünwald staining

Co-cultures of hBM-MSCs or pBM-MSCs and C2C12 myoblasts were fixed with cold methanol (− 20 °C). Then, cells were incubated with May–Grünwald and Giemsa dyes according to the manufacturer’s protocol (Merck). The fusion index of cells was calculated as the percentage of nuclei in myotubes to all nuclei visible in the field. Three independent experiments were performed.

### Statistical analysis

Results were analyzed using GraphPad Software. The mean values and standard deviations were presented, and the non-paired *t* test was performed to compare treated cells with control cells. Differences were considered statistically significant when *p* < 0.05 (marked on charts with asterisks).

## Results

### Expression of IGF-1, IL-4, IL-6, and SDF-1 and their receptors in human BM-MSCs

**Fig. 1:**
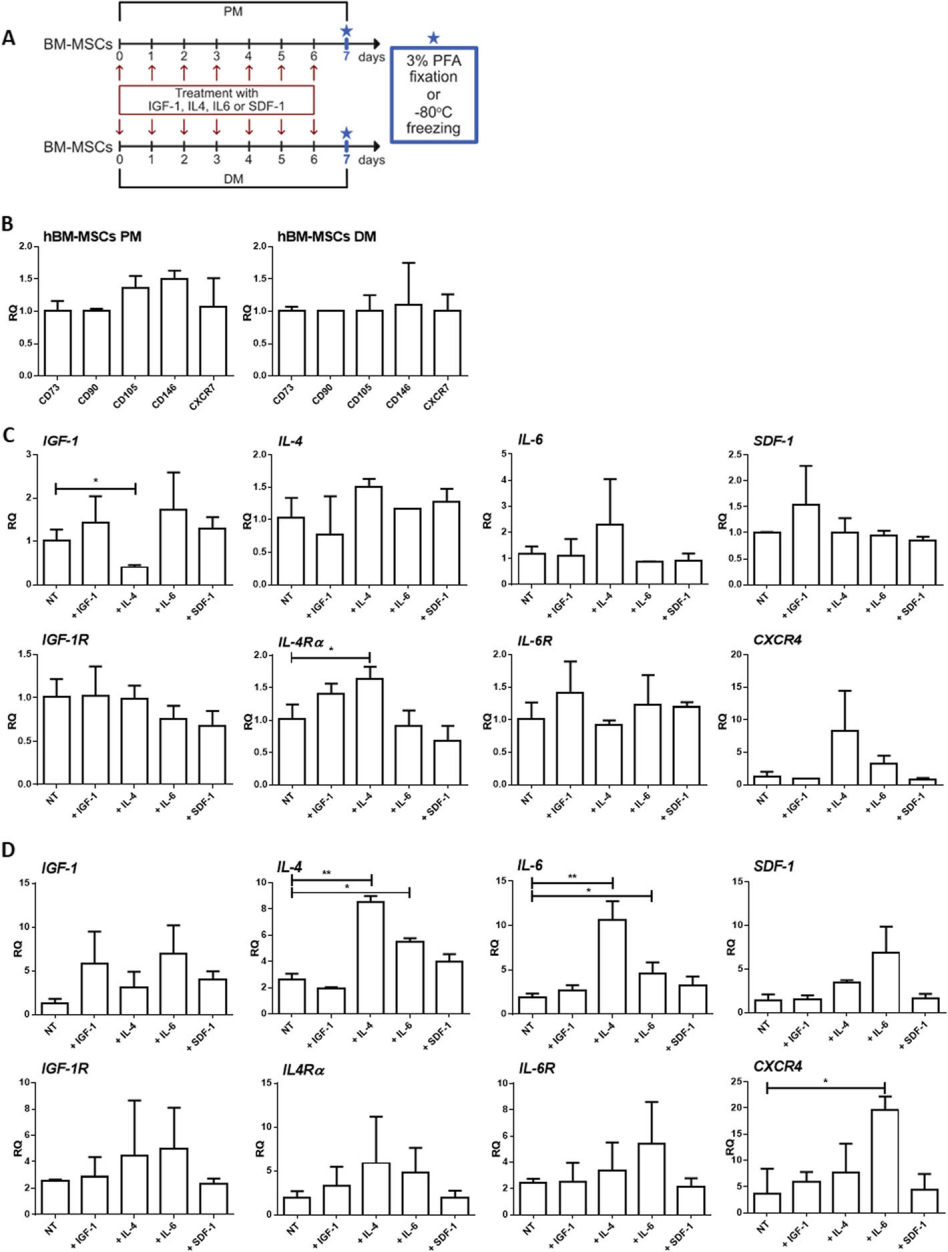
Expression of IGF-1, IL-4, IL-6, and SDF-1 and their receptors in human BM-MSCs (hBM-MSCs) non-treated (NT) and treated with either IGF-1, IL-4, IL-6, or SDF-1 cultured in the proliferating medium (PM) or differentiating medium (DM) - qRT-PCR analysis. **a** The experimental design. **b** Expression of mesenchymal stromal/stem cell markers in non-treated (NT) hBM-MSCs cultured in PM or DM for 7 days (*n* = 3). **c** Expression of cytokines and their receptors in hBM-MSCs non-treated (NT) or 7 days treated with either IGF-1 or IL-4 or IL-6 or SDF-1 cultured in PM (*n* = 3). **d** Expression of cytokines and their receptors in hBM-MSCs non-treated (NT) or 7 days treated with either IGF-1 or IL-4 or IL-6 or SDF-1 cultured in DM (*n* = 3); *p < 0.05, **p < 0.01, ***p < 0.005

In the present study we analyzed human BM-MSC cell line (further described as hBM-MSCs) according to the experimental scheme involving treatment with various factors (Fig. 1a). The cells were cultured in the presence of 50 ng/ml of either IGF-1, IL-4, IL-6, or SDF-1 for 7 days in either the so-called proliferating medium (PM) or differentiating medium (DM). Control cells consisted of non-treated hBM-MSCs. Cells analyzed at day 7 of culture were characterized by the expression of standard mesenchymal stromal cell markers, such as *CD73*, *CD90*, and *CD105* (Fig. 1b) [59]. Moreover, we confirmed that these cells expressed *CD146* that was recently described as a marker of human bone marrow-derived mesenchymal stem cells (Fig. 1b) [51]. hBM-MSCs analyzed by us also expressed both SDF-1 receptors, i.e., *CXCR4* and *CXCR7* (Fig. 1b), but the relative expression level of *CXCR4* mRNA was extremely low (data not shown).

Next, we analyzed the expression of mRNAs encoding endogenous growth factors and cytokines (IGF-1, IL-4, IL-6, and SDF-1) as well as their receptors in non-treated, i.e., control, and continuously treated cells (Fig. 1c, d). hBM-MSCs cultured in PM or DM expressed IGF-1, IL-4, IL-6, SDF-1, and IGF-1 receptor (IGF-1R), IL-4 receptor (IL-4Rα), IL-6 receptor (IL-6R), and SDF-1 receptor (CXCR4; Fig. 1c, d). Next, we compared the level of all analyzed growth factors and cytokines between non-treated cells and those ones treated with IGF-1, IL-4, IL-6, or SDF-1 and cultured in PM or DM (Fig. 1c, d). IL-4 treatment increased the expression of *IL-4* and *IL-6* in hBM-MSCs cultured in DM while in cells cultured in PM it increased the expression of *IL-4Rα* but decreased the expression of *IGF-1*. IL-6 increased *IL-4*, *IL-6*, and *CXCR4* expression in hBM-MSCs cultured in DM. Thus, IL-4 and IL-6 changed the expression of endogenous *IGF-1*, *IL-4*, *IL-6*, *IL-4Rα*, and *CXCR4* in treated hBM-MSCs but these changes were dependent of culture conditions, i.e., the presence of PM or DM.

### The proliferation and migration of human BM-MSCs in the presence of IGF-1, IL-4, IL-6, and SDF-1

**Fig. 2:**
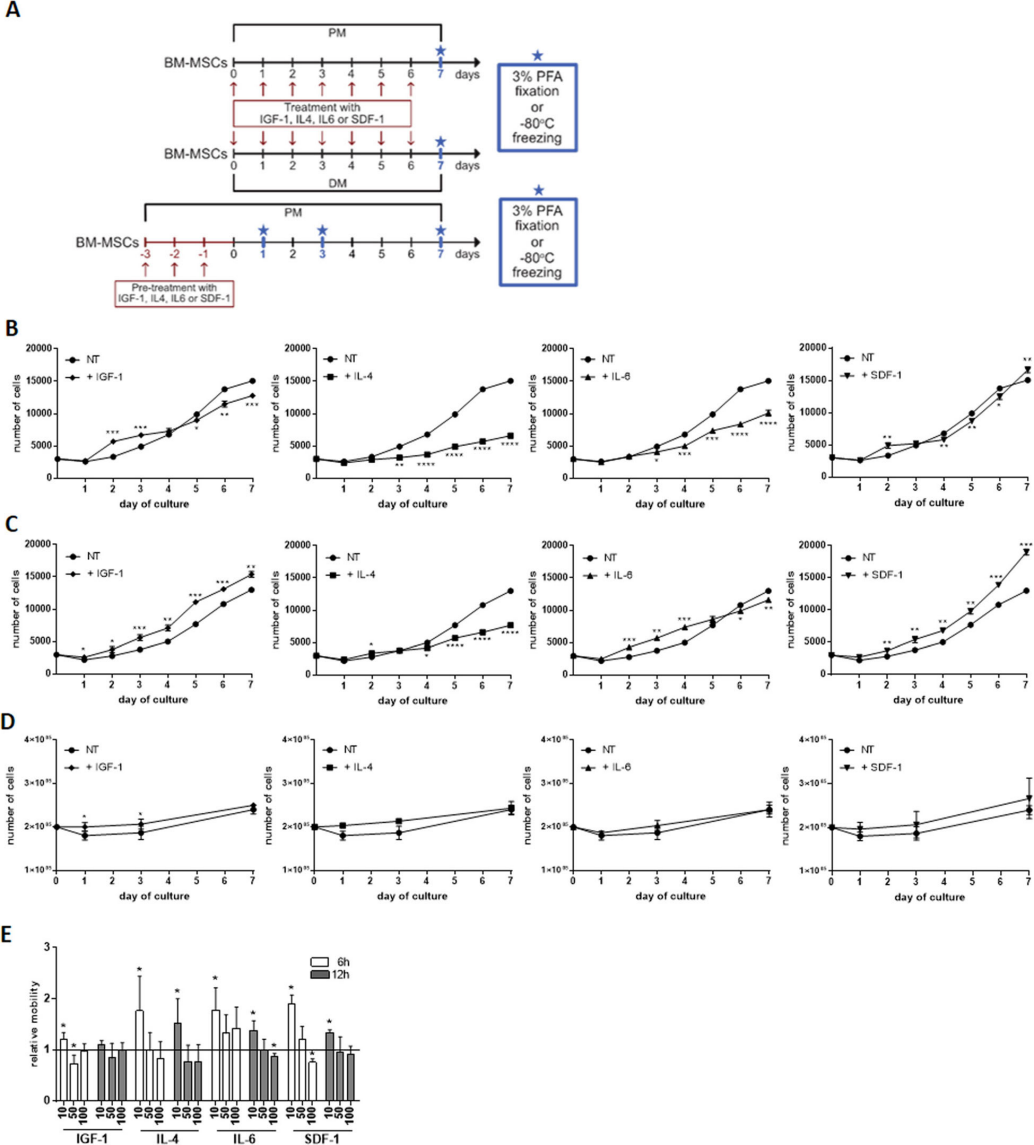
Proliferation and migration of human BM-MSCs (hBM-MSCs) treated or pre-treated with IGF-1, IL-4, IL-6, or SDF-1. **a** The experimental design. **b** The number of hBM-MSCs non-treated (NT) or treated with either IGF-1, IL-4, IL-6, or SDF-1 and cultured in the proliferating medium (PM) for 7 days (*n* = 3). **c** The number of hBM-MSCs non-treated (NT) or treated with either IGF-1, IL-4, IL-6, or SDF-1 and cultured in the differentiating medium (DM) for 7 days (*n* = 3). **d** The number of hBM-MSCs pre-treated with either IGF-1, IL-4, IL-6, or SDF-1 for 3 days and cultured in the proliferating medium (PM), analyzed after 1, 3, and 7 days (*n* = 3). **e** The migration of hBM-MSCs non-treated (NT) and treated either with IGF-1, IL-4, IL-6, or SDF-1 cultured in the proliferating medium (PM) and analyzed after 6 h and 12 h (*n* = 3). *p < 0.05, **p < 0.01, ***p < 0.005

To follow the influence of IGF-1, IL-4, IL-6, and SDF-1 on hBM-MSC proliferation, we analyzed the cell number each day during a 7-day long culture. We examined hBM-MSCs cultured according to two experimental schemes (Fig. 2a). The first set of experiments focused on hBM-MSCs treated with selected factors continuously for 7 days and then analyzed after 7 days of such culture in PM or DM. The second set of experiments focused on hBM-MSCs which were for 3 days pre-treated with the studied factors and then cultured in the proliferating medium (PM) and analyzed after 1, 3, and 7 days (Fig. 2a). In the case of continuously treated cells, their number increased regardless of the medium type used (Fig. 2b, c). After 7 days of the culture, the number of hBM-MSCs treated with IL-4 and IL-6 was significantly lower while cells treated with SDF-1 higher when compared to non-treated cells, cultured in either PM or DM (Fig. 2b, c). In the case of pre-treated cells, no significant differences were found after 7 days of the culture (Fig. 2d).

Next, we performed a scratch test to assess the hBM-MSC migration. To this point, we exposed hBM-MSCs to IGF-1, IL-4, IL-6, and SDF-1 for 6 h and 12 h. SDF-1, IL-4, and IL-6 used in the concentration of 10 ng/μl significantly increased the cell migration after 6 h as well as after 12 h compared to other tested concentrations, i.e., 50 ng/μl and 100 ng/μl (Fig. 2e). IGF-1 significantly impacted hBM-MSC migration only after 6 h (Fig. 2e). Thus, IL-4 and IL-6 significantly reduced hBM-MSC proliferation but induced their migration. Moreover, SDF-1 induced migration of the analyzed cells.

### The impact of IGF-1, IL-4, IL-6, or SDF-1 treatment on MRFs, adhesion, and structural protein expression in human BM-MSCs

**Fig. 3:**
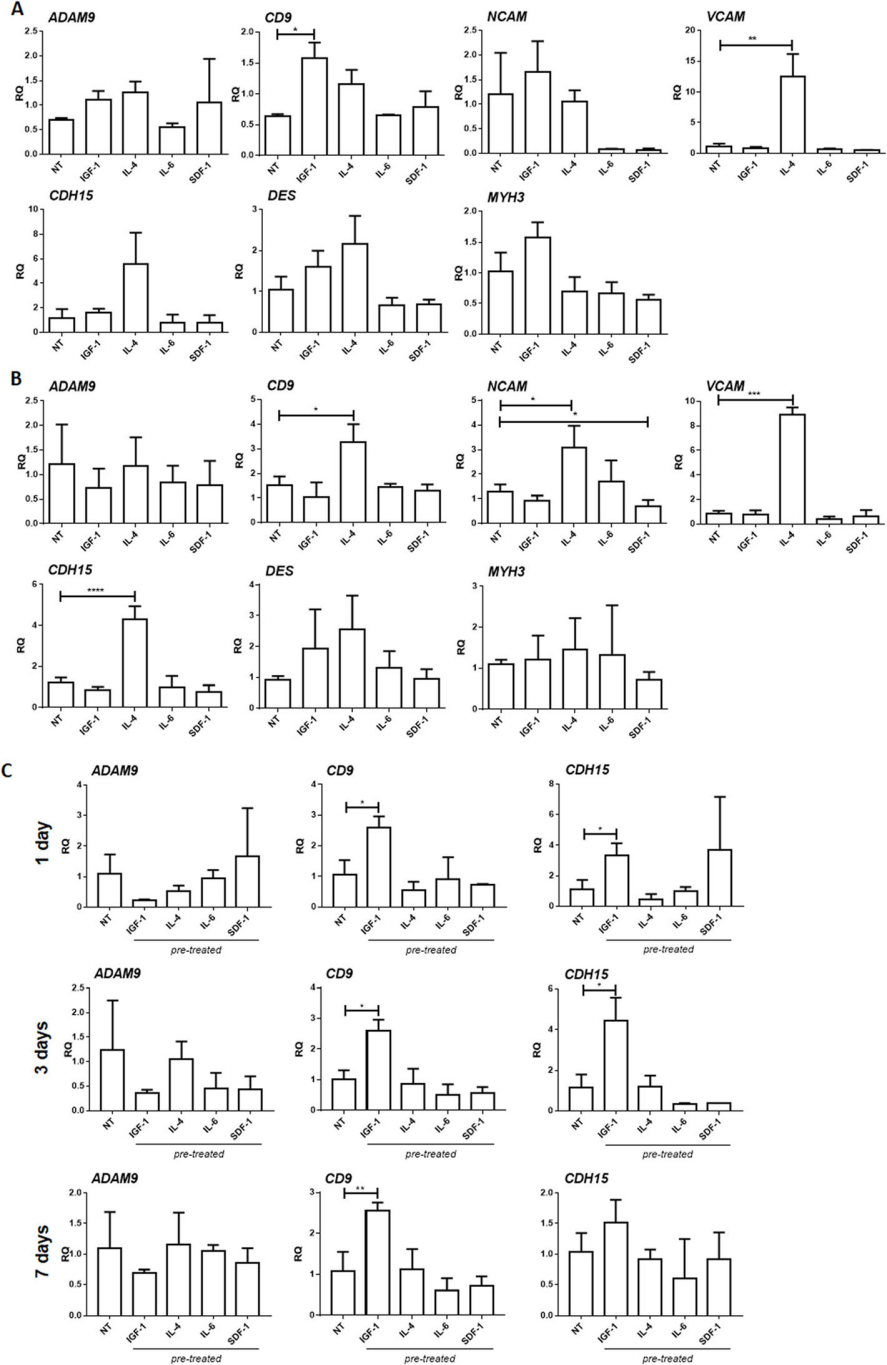
The IGF-1, IL-4, IL-6, or SDF-1 impact on adhesion and structural protein expression in human BM-MSCs (hBM-MSCs). **a** The expression of *ADAM9*, *CD9*, *NCAM*, *VCAM*, *CDH15* (m-cahderin), *desmin*, and *MYH3* (muscle embryonic myosin heavy chain 3) in hBM-MSCs non-treated (NT) or treated with either IGF-1, IL-4, IL-6, or SDF-1, cultured for 7 days in the proliferating medium (PM) (*n* = 3). **b** The expression of mRNAs encoding ADAM9, CD9, NCAM, VCAM, m-cadherin (CDH15), desmin, and MYH3 in hBM-MSCs non-treated (NT) or treated with either IGF-1, IL-4, IL-6, or SDF-1, cultured for 7 days in the differentiating medium (DM) (*n* = 3). **c** The expression of *ADAM9*, *CD9*, and *CDH15* in hBM-MSCs non-treated (NT) or pre-treated with either IGF-1, IL-4, IL-6, or SDF-1 for 3 days, then cultured in the proliferating medium (PM) and analyzed after 1, 3, and 7 days (*n* = 3); *p < 0.05, **p < 0.01, ***p < 0.005

In the next step, we analyzed if factors selected by us influence the expression of MRFs, as well as adhesion and structural protein characteristic for cells undergoing myogenic differentiation in treated hBM-MSCs (Fig. 3). First, we analyzed hBM-MSCs cultured in either PM or DM in the constant presence of IGF-1, IL-4, IL-6, or SDF-1 for 7 days (experimental scheme on Fig. 2a) but did not detect expression of *MYF5*, *MYOD*, and *MYOG* (myogenin), regardless of culture conditions (data not shown). Since MYOD, MYF5, and myogenin are involved in the specification of the muscle cell lineage [8], we concluded that hBM-MSCs were not able to follow myogenic program in response to the treatment applied by us. Then, we followed the expression of mRNAs encoding adhesion proteins that are engaged in cell adhesion, migration, and myoblast fusion, i.e., ADAM9 (disintegrin and metalloproteinase), tetraspanin CD9, NCAM (neural cell adhesion molecule), VCAM (vascular cell adhesion molecule), m-cadherin (CDH15), and structural muscle proteins, i.e., desmin and muscle embryonic myosin heavy chains 3 (MYH3). IGF-1 treatment led to a *CD9* mRNA level increase in hBM-MSCs cultured in PM (Fig. 3b) while IL-4 significantly increased expression of *CD9*, *NCAM*, *VCAM*, and *CDH15* in hBM-MSCs cultured in DM (Fig. 3b).

Next, we studied the expression of MRFs, *ADAM9*, *CD9*, and *CDH15* in pre-treated cells cultured in PM (experimental scheme on Fig. 2a). Similarly to continuously treated hBM-MSCs, pre-treated ones did not express mRNAs encoding MRFs; however, IGF-1 pre-treatment led to *CD9* and *CDH15* mRNA increase which was noticed already 1 day after the end of the pre-treatment and was observed also at days 3 and 7 (Fig. 3c). The other pre-treated hBM-MSCs did not present any significant changes in adhesion protein expression (Fig. 3c).

**Fig. 4:**
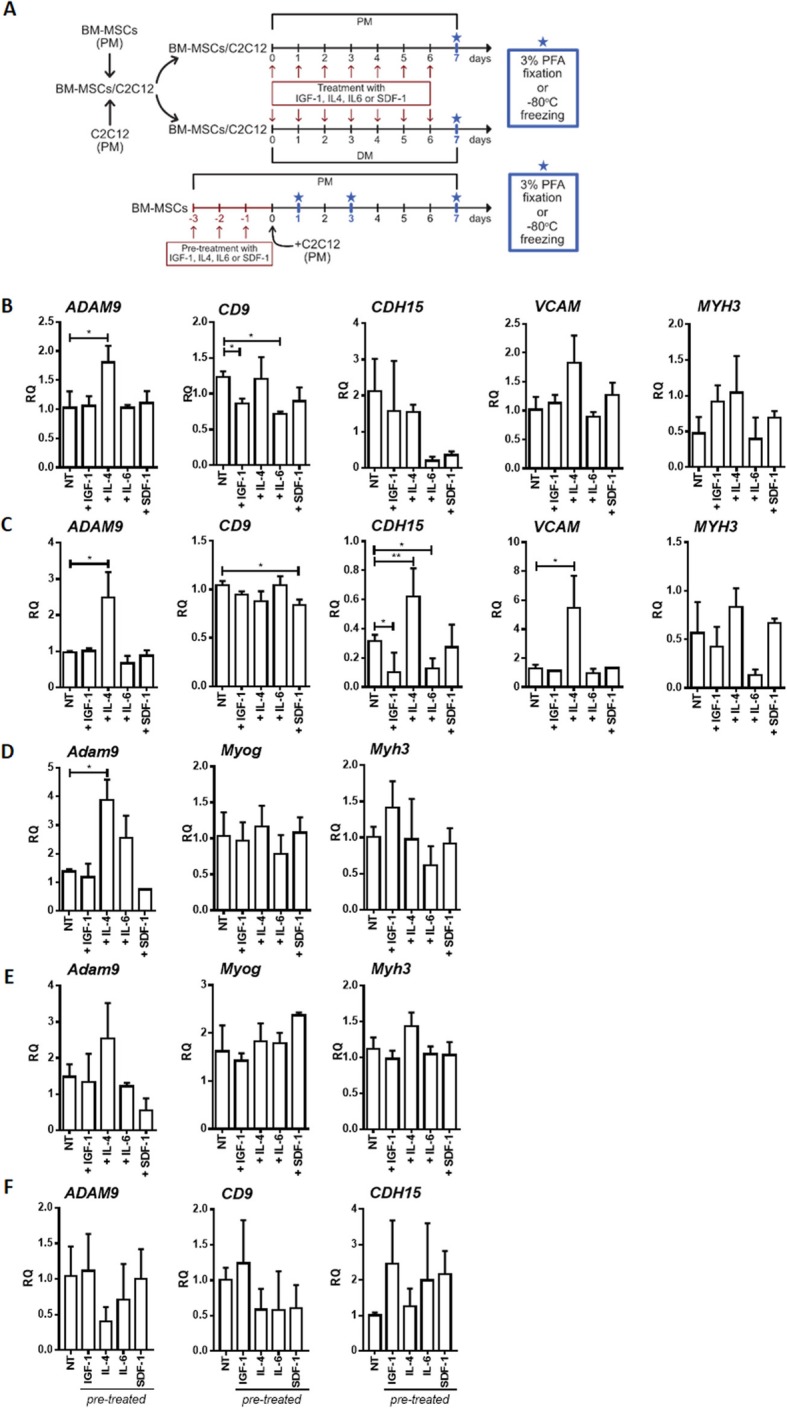
The IGF-1, IL-4, IL-6, or SDF-1 impact on MRF and structural protein expression in co-cultures of human BM-MSCs (hBM-MSCs) and mouse C2C12 myoblasts. **a** The experiment design. **b** The expression of human *ADAM9*, *CD9*, *CDH15* (m-cadherin), *VCAM*, and *MYH3* (muscle embryonic myosin heavy chain 3) in hBM-MSCs non-treated (NT) and treated either with IGF-1, IL-4, IL-6, or SDF-1 co-cultured with C2C12 for 7 days in the proliferating medium (PM) (*n* = 3). **c** The expression of human *ADAM9*, *CD9*, *CDH15* (m-cadherin), *VCAM*, and *MYH3* (muscle embryonic myosin heavy chain 3) in hBM-MSCs non-treated (NT) or treated with either IGF-1, IL-4, IL-6, or SDF-1 co-cultured with C2C12 myoblasts for 7 days in the differentiating medium (DM) (*n* = 3). **d** The expression of mouse *Adam9*, *Myog*, and *Myh3* mRNA in C2C12 myoblasts non-treated (NT) and treated with either IGF-1, IL-4, IL-6, or SDF-1 co-cultured with hBM-MSCs for 7 days in the proliferating medium (PM) (*n* = 3). **e** The expression of mouse *Adam9*, *Myog*, and *Myh3* mRNA in C2C12 myoblasts non-treated (NT) and treated with either IGF-1, IL-4, IL-6, or SDF-1 co-cultured with hBM-MSCs for 7 days in the differentiating medium (DM) (*n* = 3). **f** The expression of human *ADAM9*, *CD9*, and *CDH15* (m-cadherin) in hBM-MSCs pre-treated for 3 days with either IGF-1, IL-4, IL-6, or SDF-1 and then co-cultured with C2C12 myoblasts in the proliferating medium (PM) for 7 days (*n* = 3); *p < 0.05, **p < 0.01, ***p < 0.005

Then, we analyzed treated and pre-treated hBM-MSCs that were co-cultured with mouse C2C12 myoblasts in PM or DM. In the first set of the experiments, hBM-MSCs and C2C12 myoblasts were co-cultured in the constant presence of IGF-1, IL-4, IL-6, or SDF-1 (Fig. 4a). In the second set of the experiments, hBM-MSCs were first pre-treated with the abovementioned factors and then the co-culture with C2C12 myoblasts was established and conducted in PM (Fig. 4a). The use of mouse myoblasts allowed us to distinguish human and mouse transcripts. Again, we did not notice any significant changes in the MRF mRNA level, regardless of the treatment and co-culture conditions (data not shown). However, we observed the increase of human *ADAM9* expression level in the co-cultures treated with IL-4 and cultured in PM and *ADAM9*, *CDH15*, and *VCAM* upregulation in the co-cultures treated with IL-4 in DM (Fig. 4b, c). The level of mouse transcripts was also analyzed to verify if human IGF-1, IL-4, IL-6, or SDF-1 impacted the expression of mRNAs encoding MRFs and structural proteins in C2C12 myoblasts. Except changes in the *Adam9* level which increased in the co-cultures in PM continuously treated with IL-4, we did not detect any significant differences (Fig. 4d, e). In the co-cultures of pre-treated hBM-MSCs and C2C12 myoblasts, any significant changes in adhesion protein mRNA level between non-treated and pre-treated co-cultures were found (Fig. 4f).

### Formation of hybrid myotubes between human or porcine BM-MSCs with C2C12 myoblasts

**Fig. 5:**
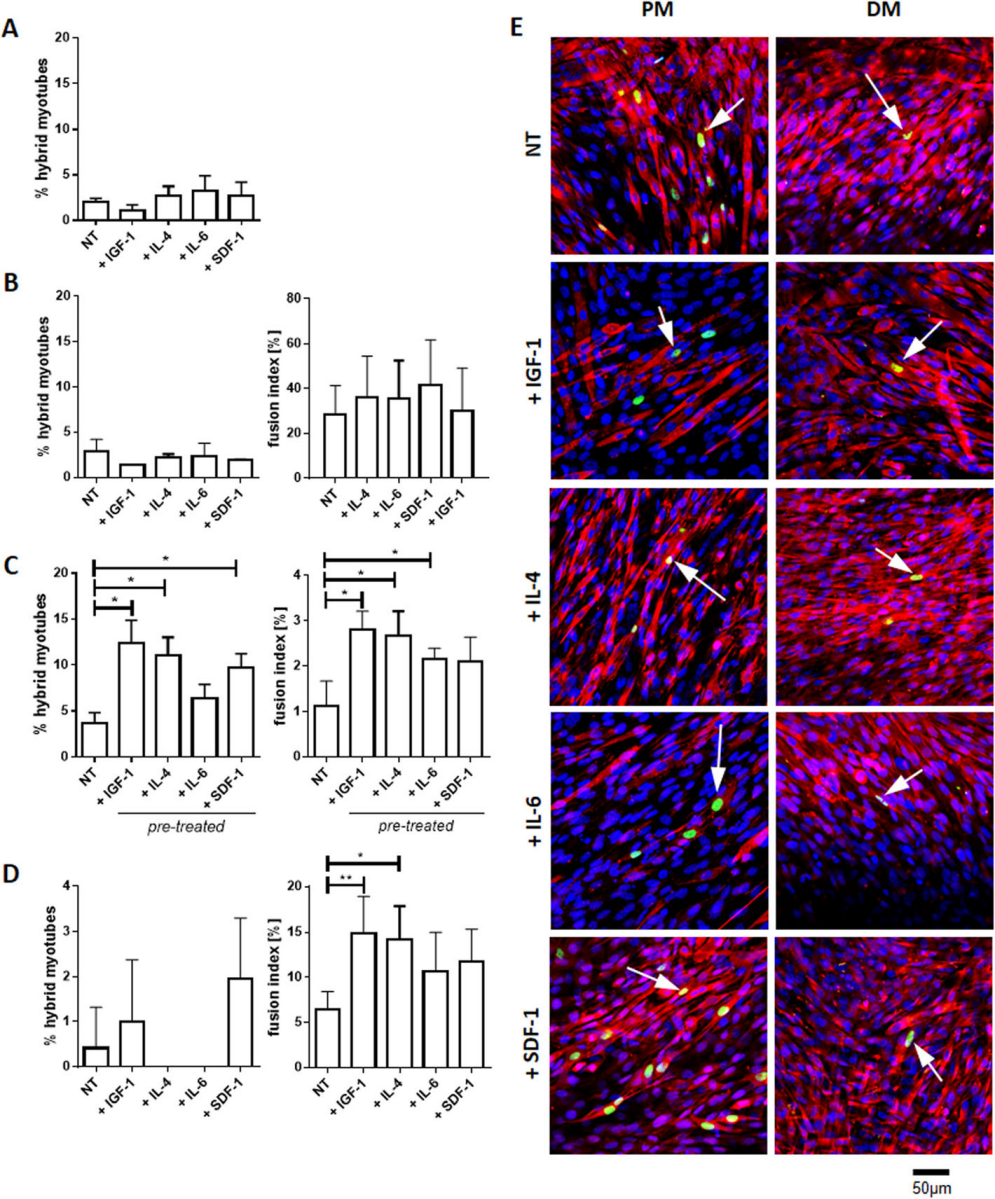
The fusion of non-treated (NT) and IGF-1-, IL-4-, IL-6-, or SDF-1-treated or pre-treated human and pig BM-MSCs (hBM-MSCs and pBM-MSCs) with C2C12 myoblasts. **a** The percentage of hybrid myotubes in co-culture of treated hBM-MSCs and C12C12 myoblasts in the proliferating medium (PM) for 7 days (*n* = 3). **b** The percentage of hybrid myotubes and the fusion index in co-culture of treated hBM-MSCs and C12C12 myoblasts in the differentiating medium (DM) for 7 days (*n* = 3). **c** The percentage of hybrid myotubes and fusion index in co-culture of hBM-MSCs and C2C12 myoblasts pre-treated for 3 days in control (without selected factor supplementation) proliferating medium (PM) for 7 days (*n* = 3). **d** The percentage of hybrid myotubes and fusion index in co-culture of non-treated (NT) or treated pBM-MSCs and C2C12 myoblasts in the differentiating medium (DM) after 7 days (*n* = 3). **e** hBM-MSC localization in myotubes in co-cultures with C2C12 myoblasts after 7 days of IGF-1, IL-4, IL-6, or SDF-1 treatment in PM and DM. Scale bar 50 μm. Blue—cell nuclei, red—skeletal myosin, green—human cell nuclei. *p < 0.05, **p < 0.01, ***p < 0.005

Our experiments showed that IL-4 or IGF-1 treatment increased expression of mRNAs encoding adhesion proteins in hBM-MSCs. Moreover, IL-4 and IL-6 influenced hBM-MSC proliferation, while SDF-1 enhanced their migration. In the next step, we tested if observed changes translated to the ability of human and porcine BM-MSCs to fuse with myoblasts. We decided to analyze porcine BM-MSCs (pBM-MSCs), in addition to hBM-MSCs, to verify if these mechanisms were species-specific. Moreover, pig already serves as an animal model in numerous studies but the understanding of porcine cell biology is limited. To this point, we analyzed co-cultures of hBM-MSCs or pBM-MSCs with mouse C2C12 myoblasts and presented the fusion index, as well as proportion of hybrid myotubes formed by human or porcine BM-MSCs and mouse C2C12 myoblasts (Fig. 5, Additional file 1: Figure S1). The use of human or porcine and mouse cells allowed to distinguish the hybrid myotubes in co-cultures. The human cells were detected on the basis of human nuclear antigen expression; porcine cells were GFP positive. Finally, myotubes were identified by myosin expression. The C2C12 cells are the ones typically used in studies involving hybrid myotube formation [51, 60]. The co-cultures were treated, i.e., cultured in constant presence with IGF-1, IL-4, IL-6, or SDF-1, or the hBM-MSCs were first pre-treated for 3 days with selected factors and then co-cultured with myoblasts in the absence of these factors (experimental scheme on Fig. 4a). Hybrid myotubes were present in both control and co-cultures treated with either IGF-1, IL-4, IL-6, or SDF-1 in PM or DM, but no significant effect of treatment was found (Fig. 5a, b, e). Similarly, the fusion index did not differ between control and continuously treated co-cultures (Fig. 5b). However, the proportion of hybrid myotubes as well fusion index was higher in the co-cultures of hBM-MSCs pre-treated with selected factors and C2C12 myoblasts, as compared to control ones (Fig. 5c). The pBM-MSCs were also able to form hybrid myotubes with C2C12 myoblasts; however, any of tested factors did not significantly impact the frequency of this process but IGF-1 and IL-4 slightly increased index of fusion in these co-cultures (Fig. 5d). Thus, the pre-treatment of hBM-MSCs with IGF-1, IL-4, or SDF-1 increased the effectivity of hybrid myotube formation by their fusion with C2C12 myoblasts.

### IL-4 treatment modifies the transcriptome of human BM-MSCs

**Fig. 6:**
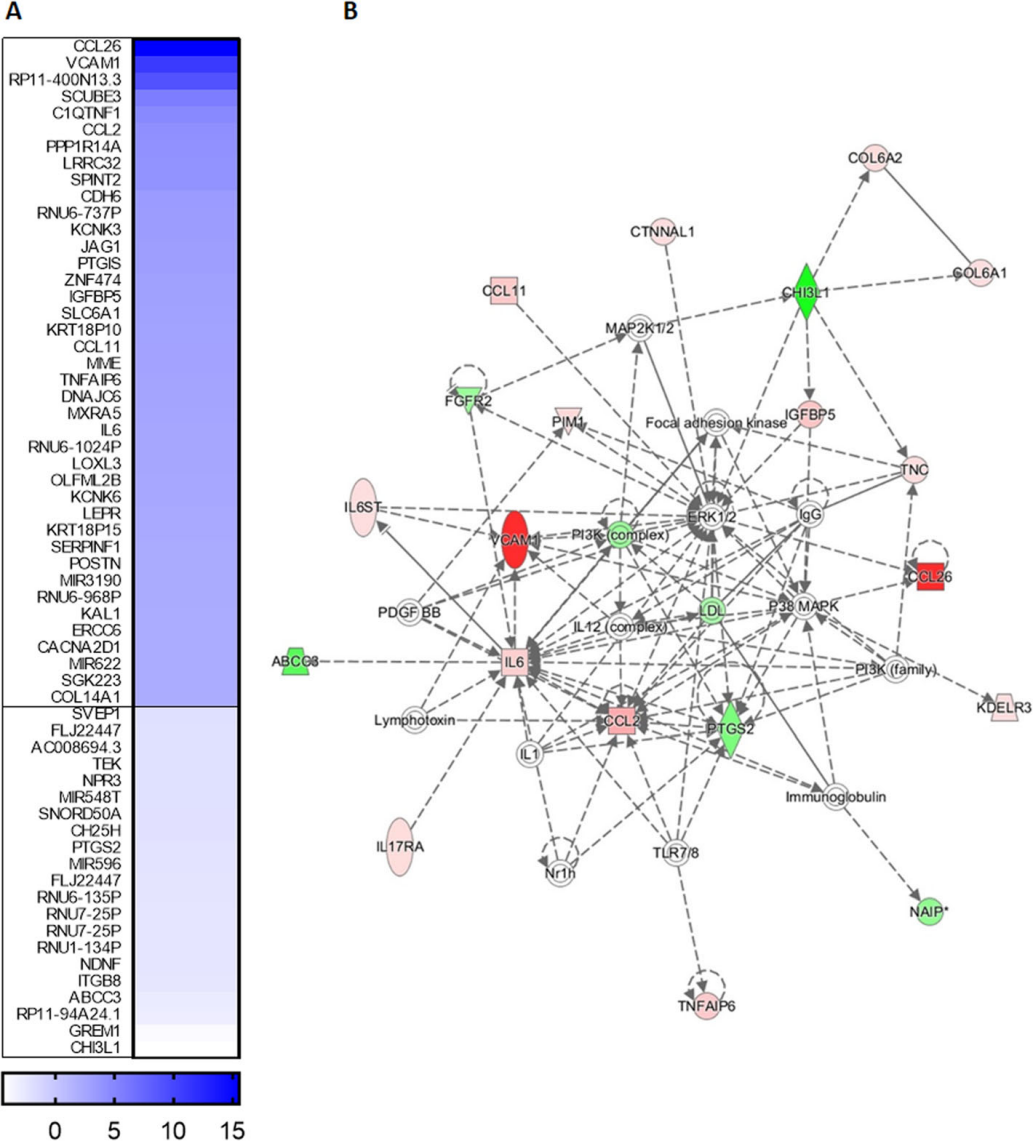
The changes in human BM-MSC (hBM-MSCs) transcriptome after IL-4 treatment. Human BM-MSCs were cultured for 48 h in the presence of IL-4 and then analyzed. **a** The changes in the level of transcripts between IL-4-treated and non-treated hBM-MSCs. **b** Gene networks created by interposing the results onto database of Ingenuity containing information about the gene function with the use of Ingenuity Pathway Analysis tool (red—upregulation, green—downregulation)

Important changes in gene expression in hBM-MSC treated with IL-4 were observed. Thus, we decided to compare the global transcriptome of control and IL-4 treated hBM-MSCs. Cells were treated for 7 days with IL-4 in PM, and then RNA was isolated and subjected to microarray analysis. Analysis of variance (ANOVA) allowed to create the lists of genes significantly upregulated or downregulated in IL-4-treated hBM-MSCs (with the cutoff values: *p* value < 0.05, − 1.3 ≥ fold change ≥ 1.3). This analysis showed that IL-4 treatment regulates the expression of 61 transcripts (Fig. 6). Using Ingenuity Pathway Analysis, we showed that IL-4 impacts the expression of many genes encoding proteins engaged in cell adhesion and migration such as VCAM (as showed also by qRT-PCR analysis), k-cadherin (cadherin-6, *CDH6*), and extracellular proteins such as collagen XIV (COL14A1; Fig. 6). The increase was also noticed in the transcript level for chemokine (C-C motif) ligand 26 and 11 (CL26, CCL11) and cytokine IL-6. Also, transcripts encoding signaling involved proteins were upregulated, among them were JAG1, i.e., membrane protein that interacts with Notch receptors, and IGFBP, i.e., insulin-like growth factor-binding protein. Summarizing, global transcriptome analysis confirmed the important role of IL-4 in the activation of adhesion and migration as well as regulation of inflammatory process and signaling in hBM-MSCs.

## Discussion

Bone marrow mesenchymal stromal cells (BM-MSCs) were first identified in mouse by Friedenstein [61, 62] and described as multipotent progenitors on the basis of their ability to proliferate, form cell colonies, and generate bone, cartilage, and adipocytes after heterotopic transplantation in vivo [63–69]. Then, the presence of multipotent stem cells in the population of human BM-MSCs was also proved. These cells expressed CD146, were perivascular located, and able to differentiate into the bone, cartilage, and bone marrow stroma [51, 70, 71]. Moreover, human CD146+ stem cells, which were isolated from heterotopic ossicles formed after subcutaneous transplantation of BM-MSCs and then cultured at clonal density, were still able to generate colonies and formed next ossicles after serial transplantation [70, 71]. Thus, human CD146+ cells isolated from BM-MSCs fulfill the criteria of multipotent stem cells, i.e., they are able to self-renew and differentiate into several cell types. Cells with similar features present in mouse bone marrow were also described on the basis of nestin expression [72].

In our experiments, we used BM-MSC cell line derived from adult human bone marrow isolated as a result of bilateral punctures of the posterior iliac crests of healthy volunteers. These cells were characterized by expression of mRNAs encoding CD73, CD90, CD105, CD146, CXCR7, and at a very low level also CXCR4 [73, 74]. Furthermore, we showed that hBM-MSCs expressed IGF-1R, IL-4Rα, and IL-6R as well as the receptors for SDF-1, i.e., CXCR4 and CXCR7. The expression of CXCR4 by BM-MSCs is discussable as the proportion of BM-MSCs found to express CXCR4 oscillates between 0 and 27% for mouse cells and 0 and 96% for human cells [75]. It was also suggested that CXCR4 expression decreased during in vitro culture and passages of BM-MSCs [76, 77]. We showed that BM-MSCs produced also mRNAs encoding endogenous cytokines such as IL-4, IL-6, SDF-1, and IGF-1. Interestingly, the expression of transcripts for IL-4Rα as well as IL-4 and IL-6 increased in IL-4-treated BM-MSCs. This corresponds to previously published data on mouse T and B cells [78].

Human BM-MSCs characterized by CD146 expression are not able to undergo spontaneous myogenic differentiation [51]; thus, these cells do not present naïve myogenic potential. Nevertheless, it was revealed that human, rat, and mouse BM-MSCs can fuse, with low efficiency, with myoblasts [52, 79]. Additionally, modification of BM-MSCs by Pax3, β-catenin, or NICD (Notch intracellular domain) overexpression can reprogram them to start myogenic differentiation and fuse with myoblasts [53–56, 79]. Recently, we showed that satellite cell niche could induce myogenic identity of BM-MSCs. Since cytokines and growth factors are an important component of the niche, we hypothesized that factors such as IGF-1, IL-4, IL-6, and SDF-1, which are involved in the regulation of myogenesis and muscle regeneration, could impact the myogenic identity, proliferation, migration, and fusion of hBM-MSCs with myoblasts.

We showed that IL-4 and IL-6 limited proliferation but promoted migration of BM-MSCs. The SDF-1 also increased migration of BM-MSCs while IGF-1 was not a strong chemoattractant for these cells. This was in agreement with the study showing that SDF-1 stimulates migration of rat BM-MSCs but IGF-1 does not [80]. To follow the impact of selected factors on the ability of BM-MSCs to form myotubes, we co-cultured them with C2C12 myoblasts. Both human and porcine BM-MSCs were able to fuse with myoblasts, however, with low efficiency. We found that pre-treatment of human BM-MSCs with selected factors, such as IGF-1, IL-4, or SDF-1, boosts their fusion with myoblasts, while adding these factors during co-culture of both cell types has no significant effect on this process. It is a very important finding as such pre-treatment is a feasible procedure, which could be used to prime BM-MSCs prior to their transplantation.

Thus, the pre-treatment of human BM-MSCs and their further co-culture with myoblasts provided better signals increasing the hybrid myotube formation than continuous treatment of analyzed co-cultures. The analysis of changes in the expression of adhesion proteins involved in cell fusion showed that numerous factors, i.e., timing of treatment with IGF-1, IL-4, IL-6, or SDF-1, and also the type of culture medium, as well as the presence or absence of myoblasts, impact the level of mRNAs encoding adhesion proteins. IL-4 treatment and culture of human BM-MSCs in the differentiating medium increased *CD9*, *NCAM*, *VCAM*, and *CDH15* mRNA expression. For cells cultured in the proliferating medium, this effect was observed only for *VCAM* mRNA. Thus, the IL-4 treatment led to *VCAM* mRNA upregulation independently of the type of the culture medium while *CD9*, *NCAM*, and *CDH15* mRNA upregulation required additional signals provided by the differentiating medium, i.e., the presence of horse serum. The increase of *VCAM* and *CDH15* expression was also noticed in the co-cultures of hBM-MSCs with myoblasts treated with IL-4 and cultured in the differentiating medium. However, these changes were not translated to the increase of hybrid myotube formation. Moreover, the presence of myoblasts and IL-4 treatment led to *ADAM9* mRNA upregulation in case of hBM-MSCs in both proliferating and differentiating media. Interestingly, the increase of *Adam9* mRNA level was also detected in C2C12 myoblasts treated with IL-4. In addition, IGF-1 treatment or pre-treatment was sufficient to increase the level of *CD9* mRNA in hBM-MSCs (cultured separately, without myoblasts) but only in cells cultured in the proliferating medium. As mentioned above IGF-1 could induce the pathways leading to myogenic differentiation [81] and enhances regeneration of injured muscles [82]. The analysis of human placental mesenchymal stromal cells showed that IGF-1 treatment and culture of these cells in differentiation medium decreased *MYOD1* expression level and did not change *MYOG* and *MYHC* level. The authors suggested that IGF-1 treatment maintains progenitor cell phenotype of mesenchymal stromal cells cultured under differentiating conditions [83]. It was also revealed that IGF-1 treatment of rat BM-MSCs did not impact at the expression level of MEF2, myogenin, alpha-sarcomeric actinin 2 (ACTN2), and desmin; however, the three-dimensional culture of rat BM-MSCs and primary myoblasts enhances their myogenic capacity [84].

Differences observed by us in the expression of mRNAs encoding adhesion proteins were translated to the ability of pre-treated hBM-MSCs to fuse with myoblasts. The cells pre-treated with IGF-1, IL-4, and SDF-1 fused more efficiently with myoblasts. This phenomenon could be connected inter alia with *CD9* and *CDH15* mRNA upregulation in IGF-1 pre-treated hBM-MSCs or increase in *IL4Rα*, *VCAM*, or *NCAM* expression in hBM-MSCs after incubation with IL-4. The improvement of hybrid myotube formation in the co-cultures of SDF-1 pre-treated hBM-MSCs with C2C12 myoblasts could be associated with their higher migration abilities, which we observed also in mouse BM-MSC and C2C12 myoblast co-cultures [49]. An important role of SDF-1 and its receptors in BM-MSC migration is well documented [47, 85–87]. Previously, we showed that SDF-1 altered actin organization via FAK, CDC42, and RAC-1 activation [47] and promoted mesenchymal stem cell, myoblast, and embryonic stem cell migration. However, treatment of hBM-MSCs with analyzed factors during co-culture with myoblasts did not result in the increase of hybrid myotube formation. This indicates that the upregulation of mRNAs encoding adhesion proteins should be induced in hBM-MSCs at early stages of cell differentiation and fusion since further treatment of cells does not increase their ability to fuse with myoblasts.

## Conclusions

Selected factors impacted BM-MSC proliferation (IL-4 and IL-6) and improved migration (IL-4, IL-6, and SDF-1). IL-4 and IL-6 changed the expression of endogenous *IGF-1*, *IL-4*, *IL-6*, *IL-4Rα*, and *CXCR4* in hBM-MSCs. IL-4 treatment resulted in the significant expression increase of *CD9*, *NCAM*, *VCAM*, and *CDH15* (m-cadherin), i.e., factors engaged in cell fusion during myotube formation. On the other hand, IGF-1 treatment led to *CD9* mRNA upregulation. However, hBM-MSCs were not able to follow myogenic program in response to any treatment applied by us. Nevertheless, the pre-treatment of hBM-MSCs with IGF-1, IL-4, or SDF-1 increased their effectivity to participate in hybrid myotube formation. Moreover, IL-4 treatment influenced the expression of cytokines that could result in inflammatory modification. Our study revealed that IGF-1, IL-4, IL-6, and SDF-1 could be important factors affecting BM-MSC adhesion, migration, and fusion; however, they could not initiate myogenic program in these cells.

## Supplementary information

**Additional file 1: Figure S1.** The hybrid myotubes in hBM-MSC and C2C12 myoblast co-cultures. Blue – cell nuclei, red – skeletal myosin, green – human cell nuclei. Scale bar 50 μm.

## Data Availability

Department of Cytology, Faculty of Biology, University of Warsaw, Miecznikowa 1 St, 02-096 Warsaw, Poland

## Abbreviations

ADAM9: A disintegrin and metalloproteinase domain-containing protein 9
ALS: Amytropic lateral sclerosis
BAD: BCL2-associated death promoter
BM-MSC: Bone marrow-derived mesenchymal stromal/stem cell
CD9: Tetraspanin CD9
CDC42: Cell division control protein 42
CDH15: M-cadherin
CXCR4: C-X-C chemokine receptor type 4 (SDF-1 receptor)
CXCR7: C-X-C chemokine receptor type 7 (SDF-1 receptor)
DM: Differentiating medium
ERK1: Extracellular signal-regulated protein kinases 1
ERK2: Extracellular signal-regulated protein kinases 2
FAK: Focal adhesion kinase
FAP: Fibroadipogenic progenitors
FBS: Fetal bovine serum
FGF-2: Fibroblast growth factor-2
GSK3: Glycogen synthase kinase 3
HGF: Hepatocyte growth factor
HPRT1: Hypoxanthine phosphoribosyltransferase 1
HS: Horse serum
IGF-1: Insulin-like growth factor-1
IGF-1R: Insulin-like growth factor-1 receptor
IL-4: Interleukin-4
IL-4Rα: Interleukin-4 receptor alpha
IL-6: Interleukin-6
IL-6R: Interleukin-6 receptor
MAPK: Mitogen-activated protein kinase
MEF2: Myocyte enhancer factor 2
MRF: Myogenic regulatory factor
MuRF1: Muscle RING-finger protein-1
MYH3: Muscle embryonic myosin heavy chain 3
MYHC: Myosin heavy chain
MYOG: Myogenin
NCAM: Neural cell adhesion molecule
NICD: Notch intracellular domain
PI3K: Phosphoinositide-3-kinase
PM: Proliferating medium
RAC-1: Ras-related C3 botulinum toxin substrate 1
SDF-1: Stromal derived factor-1
TNF-α: Tumor necrosis factor-α
VCAM: Vascular cell adhesion protein
