# Supplementary figures and images for: The factors present in regenerating muscles impact bone marrow-derived mesenchymal stromal/stem cell fusion with myoblasts
Source: Stem Cell Res Ther. 2019 Nov 21;10:343. doi: 10.1186/s13287-019-1444-1 (PMC6873517; doi:10.1186/s13287-019-1444-1)

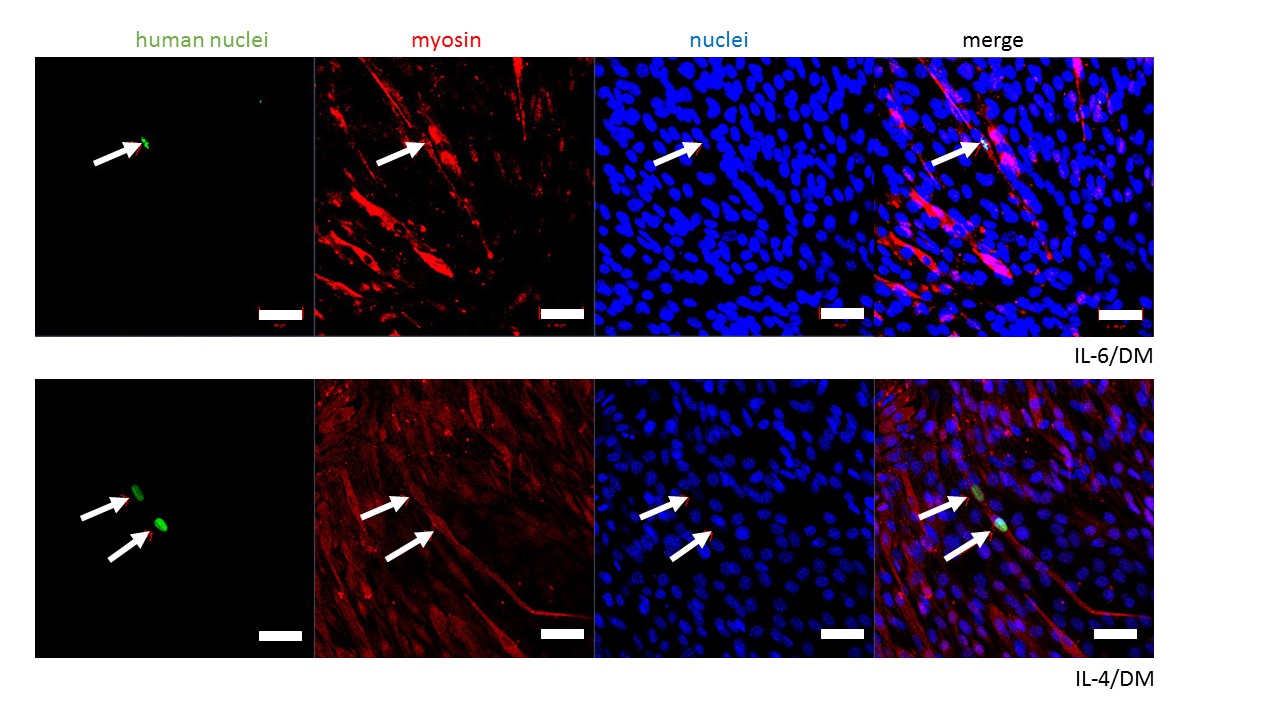

Supplement: Supplementary file 1 — Additional file 1: Figure S1. The hybrid myotubes in hBM-MSC and C2C12 myoblast co-cultures. Blue – cell nuclei, red – skeletal myosin, green – human cell nuclei. Scale bar 50 μm. [file 13287_2019_1444_MOESM1_ESM.jpg]
